# Enhanced Memory for both Threat and Neutral Information Under Conditions of Intergroup Threat

**DOI:** 10.3389/fpsyg.2015.01759

**Published:** 2015-11-17

**Authors:** Yong Zhu, Yufang Zhao, Oscar Ybarra, Walter G. Stephan, Qing Yang

**Affiliations:** ^1^School of Psychology, Southwest University, Chongqing, China; ^2^Xiayang Primary School, Xiamen, China; ^3^Key Laboratory of Cognition and Personality, Ministry of Education, Southwest University, Chongqing, China; ^4^Department of Psychology, University of Michigan, Ann Arbor, MI, USA; ^5^University of Hawaii, Honolulu, HI, USA

**Keywords:** intergroup threat, vigilance, memory bias, arousal, information

## Abstract

Few studies have examined the effect of intergroup threat on cognitive outcomes such as memory. Different theoretical perspectives can inform how intergroup threat should affect memory for threat-relevant and neutral information, such as the mood-congruency approach, Yerkes–Dodson law, Easterbrook’s theory, and also evolutionary perspectives. To test among these, we conducted two experiments to examine how exposure to intergroup threats affected memory compared to control conditions. In study 1, we manipulated symbolic threat and examined participants’ memory for threat and neutral words. In study 2, memory performance was assessed following the induction of realistic threat. Across the studies, in the control condition participants showed better memory for threat-related than neutral information. However, participants under threat remembered neutral information as well as threat-related information. In addition, participants in the threat condition remembered threat-related information as well as participants in the control condition. The findings are discussed in terms of automatic vigilance processes but also the effects of threat on arousal and its effect on information processing. This latter perspective, suggests paradoxically, that under some circumstances involving an outgroup threat, non-threatening information about outgroups can be extensively processed.

Relations between social groups are often negative, ranging from simple misunderstandings to extreme forms of violence such as genocide. It is therefore of great importance to understand the causes of these negative reactions to outgroup members. A great deal of research has gone into this effort and there is a considerable literature indicating that various factors contribute to negative intergroup relations. Some of these are: negative prior relations, realistic group conflict, social identity threat, negative stereotypes, social dominance orientation and right wing authoritarianism, ignorance, perceived and actual group differences, lack of empathy, and a host of other conditions. One factor that has begun to receive greater amounts of attention is perceptions of intergroup threat (Stephan et al., 2009).

Intergroup threats arise because members of one group (the ingroup) believe that members of another group (the outgroup) pose threats to their beliefs and values (symbolic threat) or their material welfare and safety (realistic threats). In previous research, intergroup threats (particularly intergroup anxiety) have been shown to be related to prejudice toward outgroups (Stephan et al., 1999), opposition to policies benefiting outgroups (Renfro et al., 2006; Barlow et al., 2010; Yang and Zhao, 2013), less effective communication with outgroups (Ulrey and Amason, 2001), and avoidance of outgroups (Duronto et al., 2005; Barlow et al., 2010). Some studies have also examined the effects of intergroup threat on social judgments of outgroups. These findings indicate that intergroup threat and intergroup anxiety are associated with judging outgroups as less variable (Hutchison and Rosenthal, 2011), decreased positive stereotyping (Vezzali et al., 2010), and increased perceptions of the number of work crimes committed by outgroup members (Pagotto et al., 2010).

In the present research we focus on how intergroup threat influences memory for information about the outgroup. A focus on cognitive outcomes such as memory is important because memory for information, as well as other basic processes such as categorization and attention, serve as the building blocks for judgments and decisions. For example, a focus on outcomes such as stereotyping and judgments of outgroup variability assume information about an outgroup is available in memory.

In terms of categorization processes, research has shown that stimuli are more likely to be categorized as representing outgroup members (especially outgroup males) when those targets emit signals that can be perceived as threatening, such as a masculine voice, ambiguous movements, or when they display angry facial expressions. This is especially likely for individuals who tend to perceive chronic interpersonal threats (Miller et al., 2010). Other research has shown that priming self-protective motives by having participants watch a frightening film clip resulted in categorizing male targets as racial outgroup males, especially for participants who tended to see the world as a dangerous place (Maner et al., 2012, Study 3).

Research focusing on attentional outcomes has shown that certain intergroup-relevant stimuli affect attentional processing. Trawalter et al. (2008) found that Black compared to White faces, even when just presented for 30 ms, were more likely to capture attention. Other research has shown that outgroup faces (in particular men’s) rapidly capture and hold attention. Such tendencies have been shown to interact with perceiver characteristics, for example, a tendency to see the outgroup as dangerous (Donders et al., 2008; Maner and Miller, 2013).

In terms of memory, research has shown that priming individuals’ self-protection motives (because of recently activated thoughts of being a soldier in patrol or by watching a movie where a person in being stalked) before information encoding, male and female participants displayed better memory for outgroup faces (Becker et al., 2010). Similar results have been shown on memory for angry facial expressions, an effect evinced even under constrained cognitive capacity (Ackerman et al., 2006).

So when we consider the building blocks of higher-level judgment and decisions—i.e., categorization processes, attention, and memory—research has unearthed various findings that point to a potential role of intergroup threat in shaping basic cognitive outcomes. When concerned with self-protection, individuals are more likely to categorize information as threatening, that is, if we make the reasonable assumption that outgroup males tend to be perceived as more threatening than other targets. Perceivers also allocate more attentional processing to such targets and show better memory for related information. Taken together, these findings suggest relationships between threat, the outgroup, and basic cognitive outcomes.

## Background

The present studies build upon this research foundation. Instead of focusing on the role of general threats of self-protection, for example, which could be induced by various circumstances, we focus on how threats that emanate specifically from the outgroup influence basic information processing, that is, memory for information about the outgroup.

When people feel that their group is being threatened by another group, what effects does this have on memory? This question is important because it not only reflects what information is the focus of attention during intergroup interactions, but also what information ingroup members ultimately store and use in judgments and decisions after having experienced a threatening intergroup event.

There are a variety of research traditions that are relevant to formulating hypotheses about the effects of intergroup threat on memory. The early literature on negative affect and memory suggested that memory is often skewed in the direction of affect-consistent information. That is, negative affect leads to greater memory for negative than positive or neutral information (Blaney, 1986; Mathews and MacLeod, 1994). In the current context, this literature would argue that intergroup threat should lead to better recall for negative information such as threat-related information, than for positive or neutral information—an effect that should not occur for people who are not threatened. However, there are a number of studies that suggest that while mood-congruency effects may occur for some negative emotions (e.g., depression, disgust), they are not the norm for anxiety related emotions. Specifically, for anxiety related emotions, the mood-congruency perspective is quite mixed, at times producing congruency effects (e.g., Burke and Mathews, 1992; Eysenck and Byrne, 1994), incongruency effects (Foa et al., 1989; Abalakina-Paap et al., 2001), null effects (Nugent and Mineka, 1994), or mixed effects within the same experiment as a function of memory assessed (explicit, implicit; Mathews et al., 1989). The authors of one study that found incongruency effects (better recall of positive information about the outgroup) suggest that at least in their research, the experience that was induced in participants could have resulted in modest levels of anxiety (Abalakina-Paap et al., 2001). Intergroup relations research has shown that modest levels of uncertainty and anxiety can lead to effective communication in intergroup contexts (Gudykunst, 1995). Perhaps modest levels of anxiety, which facilitate communication with outgroups, can also facilitate memory for information from intergroup contexts (Abalakina-Paap et al., 2001).

In addition to the influence of affect, some studies of the Yerkes–Dodson law (Yerkes and Dodson, 1908) have examined the effects of arousal on memory. Several of these studies indicate a curvilinear relationship (Deshpande and Kawane, 1982; Deffenbacher, 1983; Jeong and Biocca, 2012). At low levels of arousal, memory for all types of information is impeded, whereas at intermediate levels, memory is maximized. However, high levels of arousal interfere with cognitive processing and subsequent memory performance. Thus, the prediction from this approach would be that all information, both threat-related and neutral information, would be better remembered under moderate levels of arousal than when arousal levels are low or high. Consistent with this approach, one study found that moderate negative arousal increased memory for central as well as peripheral events (Laney et al., 2004). One problem with the Yerkes–Dodson law, as Deffenbacher (1994) has noted, is that there is no agreed upon metric that would enable researchers to pinpoint where on the arousal continuum an individual is.

A third literature that is relevant to predicting memory following an intergroup threat argues that arousal, in general, narrows the focus of attention to relevant stimuli (Easterbrook, 1959; McArthur and Solomon, 1978; Burke et al., 1992) and leads to better memory for relevant than neutral information. In this case, the relationship between arousal and memory is linear, the greater the arousal, the better the memory. With respect to intergroup threat, the implication is that threat-related information should be better processed and remembered than neutral information when a threatening situation has created arousal. When there is no threat, threat-related information should not be remembered any better than other types of information.

In the present research we did not induce concerns directly related to survival, nor was the to-be-processed information directly relevant to survival. However, the focus on threats to one’s future prospects (see threats used in the present studies) are relevant to issues of survival and reproduction, which makes available evolutionary-based frameworks applicable to the current theorizing. Different perspectives derived from evolutionary considerations make somewhat different predictions. Research interpreted as reflecting automatic vigilance has shown that individuals display efficient and better processing of negative over other types of information (Pratto and John, 1991). Research that has used stimuli more relevant to survival (angry faces) showed that angry faces were both more quickly and accurately detected than other faces (e.g., sad faces; Hansen and Hansen, 1988; Öhman et al., 2001), and that biologically-relevant images (sexual images, appetizing food, snake, skull) held attention longer and were better remembered than social images even under cognitive strain (Sakaki et al., 2012). However, research on adaptive memory suggests that the state of the individual (or at least the task they are performing) also matters. In incidental learning experiments, for example, participants who had to evaluate words for their survival relevance (e.g., securing food, protection from predators) remembered many more survival-related words than participants who rated the words for their pleasantness (Nairne et al., 2007).

Thus, in terms of the former perspective (automatic vigilance to threat), the hypothesis is that memory for threat–relevant information should be well remembered regardless of the state (i.e., feeling an intergroup threat) of the individual. Findings in line with this perspective indicate that faces displaying a threatening emotion were more readily noticed than happy or neutral faces (Fox et al., 2000; Eastwood et al., 2001; Pinkham et al., 2010). Also, in a shooter paradigm, the armed targets were shot at more quickly than unarmed targets (e.g., Mange et al., 2012). Threatening information seems potent and salient, as it may be generally seen as a threat to personal survival, which makes such information preferentially processed over non-threatening information. But in terms of the adaptive memory perspective, and also findings linking attention and memory to characteristics of the person (e.g., seeing the world as dangerous), the hypothesis differs and suggests that threat-related information should be best remembered when individuals experience an intergroup threat compared to individuals who do not.

## The Present Research

In order to examine the effects of intergroup threat on memory, for the present studies we created a design in which participants were or were not exposed to an intergroup threat. We then had the participants perform a memory test in which they were given threat-related and neutral information and later asked to recognize the information they had seen previously. This allowed us to determine the effects that intergroup threat had on memory for threat-related and neutral information. In the first study, the threats that were activated were symbolic ones (implying that an outgroup believed the ingroups’ values doomed them to failure). The second study was a conceptual replication of the first study in which realistic threats were activated (threats of discrimination against the participants’ ingroup).

Because the effects of intergroup threat on memory have not been previously investigated to our knowledge, there is no basis in prior research on threat that can be used to formulate hypotheses. However, the other research reviewed offers some reasonably clear hypotheses. Because we regarded the threat we presented as being moderate in intensity and because it can be classified as affectively negative, the mood-congruency approach, Yerkes–Dodson law, and Easterbrook’s theory that arousal narrows the focus of attention would suggest that there should be better memory for threat-related than neutral information after people have experienced an intergroup threat than when they have not. However, only the Yerkes–Dodson law would argue that after an intergroup threat, information that is neutral should be as well remembered as threat-related information. The automatic vigilance approach would argue that memory for threat-related information should always be superior to memory for neutral information, whereas evolutionary approaches that focus on the availability of an “adaptive” mindset or evolutionarily relevant task suggest that superior memory for threat-related information will depend on individuals experiencing a relevant (intergroup) threat. Here we assume that the experience of an intergroup threat, as operationalized in this research, has implications for an individual’s survival and ability to thrive.

## Study 1

### Materials and Methods

#### Ethics Statement

The Southwest University (China) Human Research Ethics Committee approved the present research. All participants consented to participate prior to the conduct of the research.

#### Participants and Design

Seventy-three undergraduates from Southwest university received ¥5 upon completion of the study (46 females, age range: 19–25). All participants were selected to be low SES (see footnote^1^ for selection criteria). The study took approximately 10 min to complete. Threat (symbolic threat vs. control) was manipulated between-participants; word type (neutral vs. threat) was manipulated within-participants resulting in a 2 × 2 mixed design. Thirty-eight participants were run in the symbolic threat condition and 35 in the control condition.

#### Materials and Procedure

At the laboratory participants were seated in separate experimental cubicles. All participants were informed that they would be taking part in an experiment in which the ostensible link between memory and calculation ability would be examined.

***Threat manipulation***

First, all participants were asked to read an article. Participants in the symbolic threat condition read an article, ostensibly taken from *South Weekend*—an influential newspaper in China. The article reported a story indicating that high socioeconomic status Chinese citizens consider it a waste of time for low socioeconomic members of society to go to university because it is difficult to change their status and position in life. In China, the government does not prevent low SES individuals from going to university. Actually, they encourage the participation of low SES individuals in different ways. Thus, the reported story was a direct affront on low SES individuals’ value and the belief that they can improve themselves through hard work. Participants in the control condition read an article that described the geographic location of Fujian Province. When reading the article, the participants were asked to single out specific words in the article. To ensure participants read the articles carefully, they were asked to check on a subsequent list whether 10 test words had appeared in the articles.

After reading the article, the participants completed a measure that assayed the degree of threat and negative affect experienced following the reading of the passage. This served as the manipulation check on experienced threat. For this participants completed a 6-item measure answered on 8-point scales running from 0 (*not at all*) to 7 (*very much*). The items included: threatened, angry, afraid, worried, irritated, and anxious. The items were averaged to create a composite index (*α* = 0.88), with higher scores representing the experience of greater threat and negative emotional responses.

Following the manipulation check, all participants completed a word memory task consisting of encoding, filler, and recognition phases. In the encoding phase, all participants were presented 40 words (20 neutral words, 20 threat-related words), one at a time in a random sequence at the center of a computer screen. After a fixation point appeared at the center of the screen for 500 ms, the target word was presented at the same location for 2 s, and the presentation continued until all the words were presented. In addition, we added two words at the beginning and at the end of the sequence to reduce primacy and recency effects. After the encoding phase, all participants were told to complete a mathematical calculation task for 3-min to clear working memory. Participants then completed the recognition phase, in which they saw the original 40 words in addition to 40 new words (20 neutral words, 20 threat-related words). At the center of the screen, the words appeared one at a time in random order and participants had to indicate if they had previously seen each word. The next word appeared immediately on the screen once the participant keyed a response.

The target and foil words had been previously pre-tested to ensure equivalence in valence. Pre-test participants used 7-point scales that ran from 0 (*completely not threatening*) to 6 (*completely threatening*). They also rated the words’ familiarity on 7-point scales running from 0 (*completely unfamiliar*) to 6 (*completely familiar*). A paired-samples *t*-test confirmed that the threat words were rated as more threatening (*M* = 3.51, SD = 1.28) than the neutral words (*M* = 0.81, SD = 0.80), *t*(18) = 6.81, *p* < 0.001. The familiarity of the neutral (*M* = 3.80, SD = 1.03) and threat words (*M* = 3.72, SD = 0.92) did not differ, *t*(10) = 0.45, *p* > 0.05.

After completing the recognition phase of the task, participants were thanked, compensated, and debriefed.

### Results and Discussion

#### Manipulation Check: Responses to Passages

An independent-samples *t*-test performed on participants’ responses to the passages indicated a significant difference between conditions. Confirming the manipulation of threat, participants in the symbolic threat condition reported more negative affect (*M* = 3.75, SD = 1.47) than those in the control condition (*M* = 1.71, SD = 1.46), *t*(71) = 5.94, *p* < 0.001, η^2^ = 0.332.

#### Memory Bias

Using a signal detection framework, we calculated recognition sensitivity (*d*^′^) for neutral words and threat words separately. Recognition sensitivity values were: *d*^′^ = Z_(hit rates)_ – Z_(false alarm rates)_. Hit rates were calculated by dividing the number of recognized old words by the total number of old words, while false alarm rates were calculated by dividing the number of errors on new words by the total number of new words.

**FIGURE 1:**
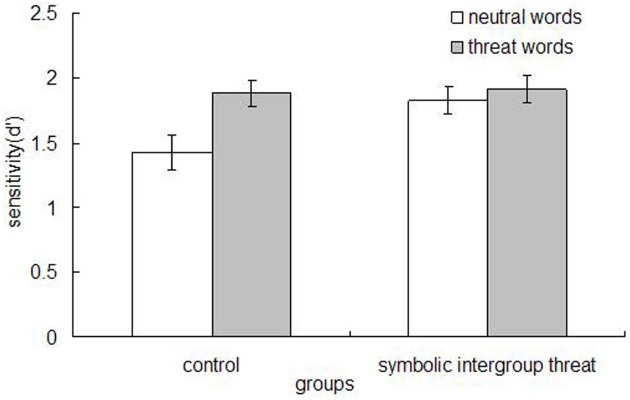
**Mean recognition sensitivity (***d***^′^) across groups, Study 1**.

The memory data (*d*^′^) were analyzed using ANOVA. The analysis yielded a main effect of word type, *F*(1,71) = 9.00, *p* = 0.004, η^2^ = 0.113, as participants recognized more threat words (*M* = 1.90, SD = 0.60) than neutral words (*M* = 1.64, SD = 0.74). More importantly, there was a significant threat condition × word type interaction, *F*(1,71) = 4.38, *p* = 0.04, η^2^ = 0.058. As depicted in Figure 1, simple comparisons indicated that participants who read the control article showed superior recognition for threat words (*M* = 1.88, SD = 0.56) relative to neutral words (*M* = 1.43, SD = 0.81), *F*(1,34) = 14.01, *p* = 001, η^2^ = 0.292. However, participants in the symbolic intergroup threat condition recognized threat words (*M* = 1.91, SD = 0.64) and neutral words (*M* = 1.83, SD = 0.63) equally well, *F*(1,37) = 0.39, *p* = 0.54, η^2^ = 0.01. Further, there was a significant difference between conditions in the degree to which participants recognized the neutral words [threat condition (*M* = 1.83, SD = 0.63) and control condition (*M* = 1.43, SD = 0.81), *F*(1,71) = 5.67, *p* = 0.02, η^2^ = 0.074]. No other effects were reliable, *F*(1,71) = 0.057, *n.s*.

Study 1 findings indicated that control participants showed sensitivity to threat-related words, similar to findings reported by other investigators (e.g., Hansen and Hansen, 1988; Pinkham et al., 2010). Specifically, they demonstrated better memory for threat compared to neutral words. However, participants in the threat condition recognized all of the words—both threat-related and neutral ones—equally well. Also, participants in the threat condition recognized more neutral words than control participants. From the different theoretical perspectives discussed in the introduction, it appears that only the Yerkes–Dodson account—given moderate levels of arousal that were presumably induced by the intergroup threat—would predict extensive processing for both the threat-related and neutral information under intergroup threat. However, to increase our confidence in these results, we conducted Study 2 to conceptually replicate Study 1 using a different type of intergroup threat.

## Study 2

### Materials and Methods

#### Participants and Design

Seventy-four undergraduates from Southwest University participated in the study and were given ¥5 upon completion of the study (42 females, age range: 18–26). All consented to participate in the study. As in Study 1, we selected participants to be low SES (see footnote 1 for selection procedure). The study took approximately 10 min to complete. Participants were assigned to either the realistic threat group (*n* = 39) or the control group (*n* = 35). Word type (neutral vs. threat) was manipulated within participants.

#### Materials and Procedure

The word stimuli and procedure were identical to those in Study 1, except that participants in the threat condition were presented with an article designed to induce realistic intergroup threat (see footnote^2^ for pilot test results confirming the difference between the symbolic and realistic threat content in the articles used in the studies). Recall that all of the participants were low SES, so in this study realistic intergroup threat was induced by having participants read a report describing how citizens of high socioeconomic status were more likely to obtain educational resources and would generally find it easier to get a better paying job compared to people of low SES. The control passage was the same one from Study 1. Following the reading of the assigned passage, participants responded to the manipulation check as in Study 1 (*α* = 0.92). Then they completed the memory task, were thanked, compensated, and debriefed.

### Results and Discussion

#### Manipulation Check: Responses to Passages

An independent-samples *t*-test performed on participants’ responses to the passages indicated a significant difference between conditions. Confirming the threat manipulation, participants in the realistic intergroup threat condition reported more threat and negative affect (*M* = 4.02, SD = 1.25) than those in the control condition (*M* = 1.64, SD = 1.49), *t*(72) = 7.46, *p* < 0.001, η^2^ = 0.436.

#### Memory Bias

**FIGURE 2:**
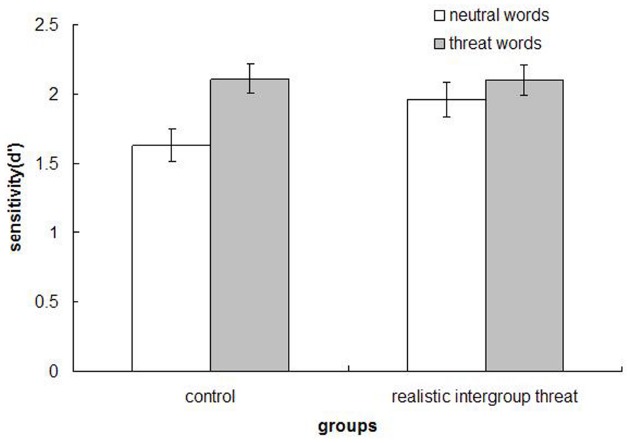
**Mean recognition sensitivity (***d***^′^) across groups, Study 2**.

Using the signal detection approach, we calculated the recognition sensitivity (*d*^′^) for the threat-related and neutral words as done in Study 1, and the memory data (*d*^′^) were analyzed using a mixed design ANOVA. The analysis yielded a main effect of word type, *F*(1,72) = 13.70, *p* < 0.001, η^2^ = 0.160, with participants better recognizing threat words (*M* = 2.11, SD = 0.66) than neutral words (*M* = 1.81, SD = 0.75). Of greater interest, there was a threat condition × word type interaction, *F*(1,72) = 4.24, *p* = 0.04, η^2^ = 0.056. As depicted in Figure 2, simple comparisons indicated that participants who read the control article showed superior recognition for threat words (*M* = 2.11, SD = 0.63) relative to neutral words (*M* = 1.63, SD = 0.71), *F*(1,34) = 16.49, *p* < 0.001, η^2^ = 0.327. However, participants in the realistic intergroup threat condition recognized threat words (*M* = 2.10, SD = 0.69) and neutral words (*M* = 1.96, SD = 0.77) equally well, *F*(1,38) = 1.37, *p* = 0.250, η^2^ = 0.035. Further, there was a marginally significant difference between conditions in the recognition of neutral words [threat condition (*M* = 1.96, SD = 0.77) and control condition (*M* = 1.63, SD = 0.71), *F*(1,72) = 3.67, *p* = 0.059 (two-tail), η^2^ = 0.048]. No other effects were reliable.

The findings from Study 2 conceptually replicated those of Study 1 using a manipulation of realistic intergroup threat. The basic type of mental processing under realistic intergroup threat appears to be the same as that induced by symbolic intergroup threat in terms of the encoding and recognition of previously processed information. Taken together, the results of the two studies strongly support the conclusion that individuals who are made to experience an intergroup threat will not only process threat-related information well but neutral information too.

## General Discussion

In the current research we used a signal detection approach, which leverages people’s ability to accurately recognize previously presented information (Green and Swets, 1966; Foa et al., 2000). The results showed that participants in the control condition better remembered threat compared to neutral words, while participants in an intergroup threat condition remembered neutral words to the same degree as threat words. The use of signal detection helps show—with good fidelity—that participants did not discriminate between the threat and neutral information when the two types of information were processed under a threatening context.

The findings for the control condition demonstrating better memory for threat-related than neutral information are consistent with an automatic vigilance perspective, which argues that people are generally vigilant with respect to threat-related information. In most situations, negative information is taken to suggest an impending threat to the individual (e.g., survival, identity, beliefs about one’s prospects), so it makes sense that such vigilance occurs even when people are not feeling directly threatened by their construal of the broader situation and circumstances (cf. Pratto and John, 1991). Related research has shown, for example, that individuals not under threatening circumstances nevertheless rapidly orient to and pay greater attention to male outgroup faces (Trawalter et al., 2008), although some research indicates this is more likely for those who associate Blacks with danger (Donders et al., 2008). Many threats in the natural environment in which humans evolved would have occurred when people were not feeling threatened, but for survival purposes such threats did need to be attended to. The findings for the general memory advantage of threat-related information thus do not accord with evolutionary perspectives on adaptive memory effects directly linked to the “mindset” (such as one involving survival) induced in individuals (Nairne et al., 2007). In the present studies, under control conditions, threat-related information was preferentially processed.

Additional evidence against the latter, evolutionary perspective comes from comparisons of memory for threat-related information between the intergroup threat and control conditions, that is, if we assume that under intergroup threat more of a “survival” mindset is active in participants. In both studies, intergroup threat did not increase memory for threat-related information. Further, the obtained findings are not as would have been predicted by the Yerkes–Dodson law or Easterbrook’s attention narrowing hypothesis. Since the participants did rate the threats as arousing, there should have been enough arousal to improve memory for threat-related information. Instead, threat-related information was well processed and remembered, regardless of whether or not the participants’ were feeling threatened.

In terms of the groups studied in this research (disadvantaged ones, although this factor was not varied in these studies), the suggestion is that low SES individuals may be chronically sensitive to SES-related information that poses a threat to their group. With respect to intergroup relations more generally, the findings suggest that there is a memory bias that may complicate matters. By attending to and remembering threat-related information, even in the absence of any current threat, people may build on their fears and negative expectations of outgroups.

Perhaps the most important finding in these studies was that under both symbolic and realistic threat there was no difference in the recognition of threat-related and neutral information. That is, not only did intergroup threats lead to high levels of memory for threat-related information, they also increased memory for neutral information. This finding is consistent only with the Yerkes–Dodson law. It suggests that the arousal created in this study was sufficient to improve memory for neutral information. This finding, though, does not fit with Easterbrook’s idea that arousal leads to a narrowing of attention to relevant stimuli since arousal-produced narrowing of attention should have led to poor memory for neutral information in the threat condition. It also does not fit with evolutionary accounts of adaptive memory that rely on activation of a relevant (e.g., survival) mindset (Nairne et al., 2007) because there is little value in recalling neutral information. Finally, these results are inconsistent with a mood priming account because any negative affect in the threat condition should not have facilitated the processing of neutral information.

There is a study in the arousal/memory literature that has obtained results that are conceptually similar to those obtained in the present studies, which suggests our findings are not an anomaly. In that study it was found that exposure to faces displaying fear (versus faces displaying neutral information) led to better memory for neutral information that was paired with fear faces than for neutral information paired with neutral faces (Davis et al., 2011).

The present studies leave unanswered the question of what the effects of threats that create high arousal would be. It is possible that at high levels of threat there would be decrements in cognitive processing. Consistent with this reasoning, two studies suggest that high levels of threat-related arousal may cause cognitive decrements. In these studies participants’ arousal was increased by inducing experiences that made them feel angry, which should have been highly arousing. In one study participants were more likely to misidentify neutral objects as threatening when they were angry than when they were not angry (Baumann and DeSteno, 2010). In the other study, anger increased mistaken judgments that target individuals possessed guns when they did not (Unkelbach et al., 2008). It would also be valuable to know if executive functioning is undermined by threats that create high levels of arousal. In particular, would people rely more on intergroup cognitive heuristics (e.g., stereotypes, the ultimate attribution error, misanthropic memory, stereotype disconfirmation bias) under high levels of arousing threat because they require fewer cognitive resources?

The finding that both neutral and threat-relevant information are well remembered when individuals experienced an intergroup threat has potentially important implications in applied settings that would be valuable to pursue in future research. For instance, this finding suggests that when intergroup threats produce modest levels of arousal people may attend to information in intergroup settings that they might otherwise not attend to or remember. This non-threat related information could be important because it could serve to humanize and individualize outgroup members and consequently influence intergroup perceptions such as the infra-humanization of outgroups and perceptions of the variability among outgroup members. That is, along with the negative effects of intergroup threat (e.g., increasing prejudice, avoidance, etc.), it is possible that intergroup threats just might have some potentially positive effects on information processing. Only additional research can answer these questions.

The present research is not without limitations. First, our manipulation-checks only used negative and not positive affect labels. It could be argued that this could have primed all participants with negative affective states (despite mean differences in reports), which could have increased sensitivity to threat-related words in both the intergroup threat and control conditions. However, it is important to point out that such an alternative cannot fully explain the pattern of results, i.e., the difference in the processing of the neutral words. Second, although in the present studies we used control conditions, it would have been useful to use a manipulation of negative affect such as anxiety (that did not involve threat), or a non-intergroup threat manipulation involving, for example, general self-protection. The memory patterns in such conditions would allow us to more accurately pinpoint the nature of the differences discovered in the present research. For example, if after a negative affect (anxiety) induction the memory patterns were found to be dissimilar to the intergroup threat conditions, our conclusions could more strongly speak to the role of intergroup threat versus anxiety in information processing. In addition, a non-intergroup threat condition would allow us to state with greater confidence that intergroup threat *per se*, and not just any threat, is what is driving the present findings. Finally, and related to the threats used in this research, a limitation is that the threats were focused at the group level, but in the intergroup threat literature a distinction is made between threats that are aimed at the ingroup as a whole and threats that are aimed at individuals because they are members of this group (Davis and Stephan, 2011). In fact, Davis and Stephan found that individual and group threats had different effects on emotions. Group threats produce more anger than fear whereas individual threats had the opposite effect. It would be interesting to see if the pattern of results that were found in the present studies will extend to threats that are more individualized. Assuming a moderately strong covariation between fear and anxiety (e.g., Sarnoff and Zimbardo, 1961), the group versus individualized threat distinctions could also help tease out the effect of anxiety in the processing of intergroup-related information.

## Conclusion

Different research findings point to relations among threat, intergroup perception, and basic cognitive processing. But to our knowledge the present research provides the first memory evidence concerning how people process threat-related and neutral information under intergroup threat. The results revealed that participants, regardless of condition, remember threat-related information well, which we argue stems from such information always being important and personally relevant. But intergroup threat, whether it was of a symbolic or realistic nature, also increased memory for neutral information apparently because the arousal created by threat improves the cognitive processing of such information.

### Conflict of Interest Statement

The authors declare that the research was conducted in the absence of any commercial or financial relationships that could be construed as a potential conflict of interest.

## Footnotes

1 Family socioeconomic status selection measure: we developed a 5-item measure to assess family SES, including parents’ education and profession as well as family’s monthly income. We used parent instead of student information because college students typically are not financially independent and will therefore have a status based on their upbringing in their family’s household (Åslund et al., 2009; Henry, 2009). Among 7 contiguous categories ranging from 1, little or no literacy; 2, primary school; 3, middle school; 4, high school; 5, junior college; 6, undergraduate; 7, postgraduate, participants chose one on behalf of their father or mother’ education—the one with the highest education level. Then they responded to a question on parents’ occupation. Participants selected from 1, unemployed/migrant worker/temporary worker; 2, manual worker; 3, skilled worker; 4, office worker; 5, common managerial/technical personnel; 6, middle-level managerial/technical personnel; 7, high-level managerial/technical personnel. Participants also responded to “What do you estimate your parents’ combined monthly income to be?” with a choice of 7 options ranging from 1 = under ¥600, to 7 = ¥12000 or over. The options were scaled in increments of ¥1200 (e.g., ¥600–¥1799, ¥1800–¥2999) up to ¥2999, beyond which the scaling changed to increments of ¥3000. The median response was 4 = ¥3000–¥6000. Participants were categorized as low SES if there total score was less than 10 (Henry, 2009).

2 To ensure that the symbolic intergroup threat article was related to symbolic threat rather than realistic threat, and that the realistic intergroup threat article was related to realistic threat rather than symbolic threat, we conducted a pretest with 20 participants who were given definitions for both symbolic and realistic intergroup threat. Then they read the two articles and rated the two articles on how related they were to the two types of threat on scales that ran from 0 (*not at all*) to 6 (*very much*). The paired-sample *t*-test showed that the symbolic intergroup threat article was rated more related to symbolic threat (*M* = 5.05, SD = 1.45) than realistic threat (*M* = 2.95, SD = 1.50), *t*(19) = 4.27, *p* < 0.001. The realistic intergroup threat article was rated as more related to realistic threat (*M* = 4.10, SD = 1.29) than symbolic threat (*M* = 2.60, SD = 1.79), *t*(19) = 2.48, *p* = 0.023. At the same time, the symbolic intergroup threat article was rated as more related to symbolic threat (*M* = 5.05, SD = 1.45) than the realistic threat article (*M* = 2.60, SD = 1.79), *t*(19) = 4.85, *p* < 0.001. The realistic intergroup threat article was rated as more related to realistic threat (*M* = 4.10, SD = 1.29) than the symbolic threat article (*M* = 2.95, SD = 1.50), *t*(19) = 2.21, *p* = 0.039.
